# Listen

**DOI:** 10.1007/s11606-026-10327-4

**Published:** 2026-03-09

**Authors:** Ian Daniel Millstein

**Affiliations:** Cooper Medical School of Rowan University, Camden, NJ USA

**Keywords:** communication, reflection, hospice

He is 80 years old with stage IV liver cancer and was hospitalized after suffering an immune reaction to his chemotherapy. A quick review of the medical record confirms that multiple rounds of chemotherapy have yielded poor results. My preceptor asks me to interview him and assess his condition since recently returning home from the hospital.

I enter the exam room and am greeted by a weary-looking man in a wheelchair. He barely resembles the photo in his chart. The vibrant older man in the image, taken just a year earlier, now appears drawn and frail, a striking change in just a short time.

Just a few weeks into my first medical school clinical rotation at an outpatient primary care site, I feel nervous broaching this patient’s difficult journey with cancer and the heavy emotions that are likely to come along. At this stage in my training, I’m not sure I am ready for the challenge of initiating a serious illness conversation that, until now, I have only learned about in lectures. Will he be open to talking to me, a 24-year-old medical student with far less experience than his regular care team? Thankfully, one particular mentor’s adage surfaces in my thoughts—*when you’re not sure what to say, focus on listening.*

I begin the visit by inviting my patient, who was accompanied by his daughter, to share his understanding of his condition and current treatment status. He speaks freely, acknowledging that his long-term prognosis is poor and that his body has failed to respond to treatments. Still, he expresses a desire to pursue another round of chemotherapy once he regains strength and weight following his recent hospital stay.

As he goes on, my shoulder muscles start to relax, and my breathing slows. He relays what he has heard of the treatment pros and cons, and I eventually ask him if his goals at this point are more focused on maximizing his remaining time or prolonging his life as long as possible.

His daughter adds her thoughts: “It feels like being in the hospital for so long took away a lot of your strength and seemed to hurt more than it helped,” she says, a somber expression mirroring her words. He mentions that his doctors discussed hospice care with him while in the hospital, but he declined. “Hospice is like giving up,” he says. He feels he has no reasonable option other than to keep fighting with whatever strength he has left.

Listening to their exchange helps me recognize what has not yet been said, creating the opening for a more discerning question, one shaped by a fuller understanding of how he views both his illness and the idea of hospice.

“How would you feel if you restarted chemo, but ended up back in the hospital again?” I ask.

“I wouldn’t want that,” he replies calmly but firmly, taking a moment to reflect before responding. In that moment, I realize how a simple question can shift the conversation from abstract fears to the concrete realities he is weighing. I give the patient and his daughter some time alone while I step out to discuss the situation with my preceptor. A few minutes later, we re-enter the exam room together.

My preceptor gives a warm greeting, listens patiently, and acknowledges the gravity of this decision about hospice care.

“Let’s review what you have been through with the cancer so far,” he says. They move through the milestones of his treatment, ending with the hospitalization that marked a turning point. He had started his latest round of chemotherapy hoping for a meaningful response, but the course was cut short after a sudden hypersensitivity reaction, and he soon found himself back in the emergency department. The multi-week admission that followed drained the strength he had rebuilt after his first hospitalization.

During that stay, a constant rotation of specialists, shifting plans, and repeated discussions about hospice made it difficult for him to feel anchored in the course of his own care. Weak from the prolonged admission, he was met with mixed messages that blurred the line between routine updates and discussions about how limited his options had become. Now, in clinic, he says he wants a clearer sense of what the path forward could look like so he can set goals that make sense for where he is now.

“Tell me about what you would definitely not want to do as your illness worsens.”

He says he wants to avoid severe pain like he experienced during his last admission, another lengthy hospitalization, and interventions such as intubation or CPR that could extend his life without meaningfully improving it. What comes next surprises me.

“I wish there was a way to cure your cancer. Since unfortunately there is none, we will help you avoid unnecessary suffering. You did not use the word *hospice*, but you described exactly what they do.”

The patient and his daughter’s eyes meet, and they seem to exchange an unspoken understanding. My preceptor suggests a short-term follow-up status check visit. We both wish him well as they leave the office.

Several weeks later, I learn that the patient eventually accepted hospice care and passed away peacefully at home.

As a medical student, I am struck by the power of presence—the simple yet profound act of listening carefully and patiently to someone’s story of illness. In one particular moment, I saw how this relieved tension not only for the patient but also for his daughter and even for me.

The experience reminds me that while clinical medicine is filled with complexities—diagnoses to uncover, questions to ask, and answers to analyze, listening is the foundation upon which all of these skills are built. It is also a segue to more constructive, insight-driven interview questions. My history and physical template is packed with prompts guiding what information I should gather, but it’s clear to me now that the heart of this process lies in how well I listen.

